# Perspectives on the essential skills of healthcare decision making in children and adolescents with intellectual disability

**DOI:** 10.1186/s12939-024-02204-5

**Published:** 2024-06-07

**Authors:** Jenny Downs, Jessica Keeley, Rachel Skoss, Jaquie Mills, Thom Nevill, Alice Schippers, Olivia Lindly, Sandra Thompson

**Affiliations:** 1 Centre for Child Health Research, Telethon Kids Institute, University of Western Australia, Perth, Australia; 2 Curtin School for Allied Health, Curtin University, Perth, Australia; 3 Institute for Health Research, University of Notre Dame, Fremantle, Australia; 4 School of Population and Global Health, University of Western Australia, Perth, Australia; 5 Microboards Australia, Perth, Australia; 6 Disability Studies, Department of Care Ethics, University of Humanistic Studies, Utrecht, the Netherlands; 7 Department of Health Sciences, Northern Arizona University, Flagstaff, AZ USA; 8 Western Australian Centre for Rural Health, University of Western Australia, Geraldton, Australia

**Keywords:** Intellectual disability, Health literacy, Shared decision making, Supported decision making, Disability Royal Commission

## Abstract

**Background:**

Involvement in healthcare decisions is associated with better health outcomes for patients. For children and adolescents with intellectual disability, parents and healthcare professionals need to balance listening to a child’s wishes with the responsibility of keeping them safe. However, there is a scarcity of literature evaluating how to effectively involve them in decision making. In this context, we review the concept of health literacy, focusing on the skills of healthcare decision making for children and adolescents with intellectual disability.

**Methods:**

We describe the concept of health literacy and models explaining shared decision making (individuals and healthcare professionals collaborate in decision making process) and supported decision making (when a trusted person supports the individual to collaborate with the healthcare professional in the decision-making process), and a rapid review of the literature evaluating their efficacy. We discuss healthcare decision making for children and adolescents with intellectual disability in the context of relevant recommendations from the recent Disability Royal Commission into Violence, Abuse, Neglect, and Exploitation of People with Disability in Australia.

**Results:**

Health literacy skills enable individuals to access, understand, appraise, remember and use health information and services. Shared decision making has been described for children with chronic conditions and supported decision making for adults with intellectual disability. Decision-making contributes to how individuals appraise and use healthcare. The rapid review found very limited evidence of outcomes where children and adolescents with intellectual disability have been supported to contribute to their healthcare decisions. Recommendations from the Disability Royal Commission highlight current needs for greater efforts to support and build the capacity of individuals with disability to be involved in the decisions that affect their life, including healthcare decision making.

**Conclusions:**

Existing rights frameworks and healthcare standards confirm the importance of providing all people with the opportunities to learn and practise health literacy skills including decision making. There is little literature examining interventions for healthcare decision making for children with intellectual disability. Childhood is a critical time for the development of skills and autonomy. Evidence for how children and adolescents with intellectual disability can learn and practice healthcare decision-making skills in preparation for adulthood is needed to reduce inequities in their autonomy.

**Supplementary Information:**

The online version contains supplementary material available at 10.1186/s12939-024-02204-5.

## Background

The adoption of the United Nations Convention on the Rights of the Child [1] confirmed children’s status as human rights holders and shifted the way children are perceived, from objects in need of protection to subjects of rights with agency, emerging capabilities and rights. The principle of Gillick competence is legally recognised and refers to children younger than 16 years who have the competence to consent to a healthcare procedure without parental involvement, providing they can demonstrate sufficient maturity and ability to appraise the proposed treatment, its risks and alternative courses of actions [2]. However, children’s intellectual immaturity or developmental stage mean they may need to rely on adults. It is increasingly recognised that children have agency, evolving capacities and emerging autonomy, and that children want to and can participate in decision-making that affects them, when they are supported to do so as they grow and mature. The United Nations Convention on the Rights of the Child and the principle of Gillick competence help people who work with children to balance the needs of listening to children’s wishes with the responsibility to keep them safe.

People with intellectual disability experience difficulties with conceptual, social, and practical adaptive skills [3]. People with intellectual disability have many strengths and positive wellbeing is observed when the child is in good physical and mental health, has opportunities to interact with family and community members, participates in a variety of activities, and is working towards achieving autonomy in daily tasks [4–7]. Alongside, the children live with difficulties in developmental and adaptive functioning domains including communication, motor, social and daily living skills. The high prevalence of physical and mental health issues may also affect their wellbeing [8–10]. For example, many children with intellectual disability live with sleep disturbances [11]. Other comorbidities vary by the underlying cause of intellectual disability. For example, children with Down syndrome have low rates of epilepsy (approximately 6%) [12] whereas epilepsy is highly prevalent in children with genetically caused epilepsy disorders which are associated with more severe disability [13]. Children with intellectual disability have greater risk of hospitalisation, 2 to 10 times greater than the general paediatric population, depending on the severity of intellectual disability [14]. Evidence from Canada and Australia shows that many of these hospitalisations are potentially preventable (such as for vaccine preventable pneumonia) compared to children without intellectual disability [15, 16]. Health problems [17] and high rates of potentially preventable hospitalisations persist into adulthood [15, 16].

Approximately 3% of children globally are affected by intellectual disability [18]. They have greater exposure to the social determinants of health, including poverty, unemployment, exposure to discrimination and violence, and barriers in accessing effective healthcare [19]. Access to healthcare services is influenced by factors at multiple levels [20]. For example, healthcare services need to be known about by relevant individuals and families, able to meet healthcare needs in a culturally appropriate way, reached physically in a timely manner and affordable [20]. In turn, individuals and their families need to understand their healthcare needs, access, engage with and be able to afford appropriate services [20]. Access to healthcare services is part of the broader concept of health literacy, which refers to the multiple skills needed by individuals, clinicians and service providers to enable effective use of health information and services [21].

In response to community concerns about disadvantage experienced by people with disability in Australia, the Australian Government established a Disability Royal Commission into Violence, Abuse, Neglect and Exploitation of People with Disability in April 2019. This wide-ranging investigation collected evidence directly from people with disability and community-wide stakeholders, across education and workplace, justice, accommodation, day program and healthcare settings. Stark inequities were exposed. The final report was published in September 2023, and contains 222 recommendations of how Australia could be a more inclusive and just society for people with disability (https://disability.royalcommission.gov.au/publications/final-report). Volume 6 of the Disability Royal Commission report, titled ‘Enabling autonomy and access’, has many recommendations that directly reflect the concepts of health literacy, including healthcare decision making.

We undertook a rapid review to evaluate the efficacy of decision-making interventions for children and young people with intellectual disability; the dearth of literature prompted this paper. In this paper, we discuss (1) the concept of health literacy and then focus on healthcare decision-making, drawing on literature relating to children (without intellectual disability) and adults with intellectual disability to inform understanding for children with intellectual disability; (2) relevant recommendations in the final report published by the Royal Commission into the Violence, Abuse, Neglect and Exploitation of People with Disability in Australia [22]; and (3) recommend future directions for healthcare decision-making research for children and adolescents with intellectual disability.

## Health literacy and healthcare decision making

### Health literacy

#### Health literacy skills are a driver of good health

Individuals need health literacy skills to access, understand, appraise, remember and use health information and services [21, 23–25]. Service providers need to be able to recognise and support health literacy needs, the strengths and preferences of individuals and caregivers, at the levels of clinical care, systems planning, and policy settings [26].

People are better positioned to make effective healthcare decisions when they understand the factors that influence their health and how to navigate and appraise needed information and services. Health literacy skills enable individuals to make meaningful contributions to their health and healthcare, with implications for health service use, outcomes, cost, and equity [27, 28] and predicting health and health outcomes [23, 29]. In Australia, the National Safety and Quality Health Service Standards recommend that individuals should have an active role in their healthcare, their health information needs should be met, decision making should be shared (between consumers and clinicians), and consumers should participate in developing their healthcare actions [30]. These standards are consistent with the notion of person-centred care, where the perspective and values of the individual are prioritised in healthcare delivery [31].

#### Decision making is a critical component of health literacy

Decision making has been described along a continuum, ranging from complete autonomy where the individual makes their healthcare decisions entirely on their own, to beneficence where the health professional or caregiver is exclusively responsible for any final decision while acting in the best interest of the patient [32]. Healthcare decisions apply to assessments, treatments, care and supports [32]. Most people need assistance to make at least some healthcare decisions, irrespective of the presence of intellectual disability [33].

Healthcare decision making involves being able to access and understand the relevant medical information and weigh up available options including potential impacts and risks before a decision is made [34]. Capacity to make a decision about healthcare requires accessible information (e.g., presented in language and format that is understood), discussion with trusted others, adequate time to consider options, understanding potential risks, and having had opportunities to develop and practice healthcare decision-making skills, irrespective of the presence of intellectual disability.

#### Healthcare decision making by children

The Convention on the Rights of the Child states that children and their families need access to information that can support healthy behaviours and choices [1]. Best practice in paediatric care has long identified the need for greater involvement of children in their healthcare decision making [35].

Decision making competence begins to develop soon after early childhood [36] and increases with the child’s development in language and communication, reasoning, and abstract thinking [37]. The child has evolving competencies as they mature, and needs support and guidance from adults to become competent decision makers for their own healthcare [38]. Whilst parents and healthcare professionals are involved in decision making for most aspects of children’s healthcare, there is more variable involvement of the child. For example, a Swedish observational study of healthcare interactions in hospital settings found inconsistent participation of 32 2- to 18-year-old children (including some children with intellectual disability) where child involvement in decision making varied within age groups irrespective of the presence of intellectual disability [39].

Children’s capacity to make healthcare decisions varies by cognitive capacity, the type of healthcare decision, available support, and previous opportunities for their practice and learning. The development of partnerships between children, parents and healthcare professionals in the process of healthcare decision making, titrated to individual contextual factors including whether or not the child can or wants to be involved in decisions about their healthcare, is critical to person-centred care [35].

#### Healthcare decision making by people with intellectual disability

The United Nations Convention on the Right of Persons with Disability (UNCRPD, 2006) clearly documents the right for people with disability to make decisions freely and exercise their autonomy [40]. Accordingly, children with intellectual disability have rights to learn health literacy skills, developing their ability to understand and use health information and contribute to decisions relating to their healthcare. This could facilitate the development of optimal autonomy in healthcare when an adult.

Healthcare decision making by individuals with intellectual disability is increasingly recognised in practice, policy, and legal settings [33]. It is important to note that the onus for understanding and making healthcare decisions should not be placed exclusively on the individual with intellectual disability. Service providers must ensure that individuals with intellectual disability and their families are provided with accessible information and support and enable meaningful communication to inform their decision making.

### How to facilitate healthcare decision making

**Fig. 1:**
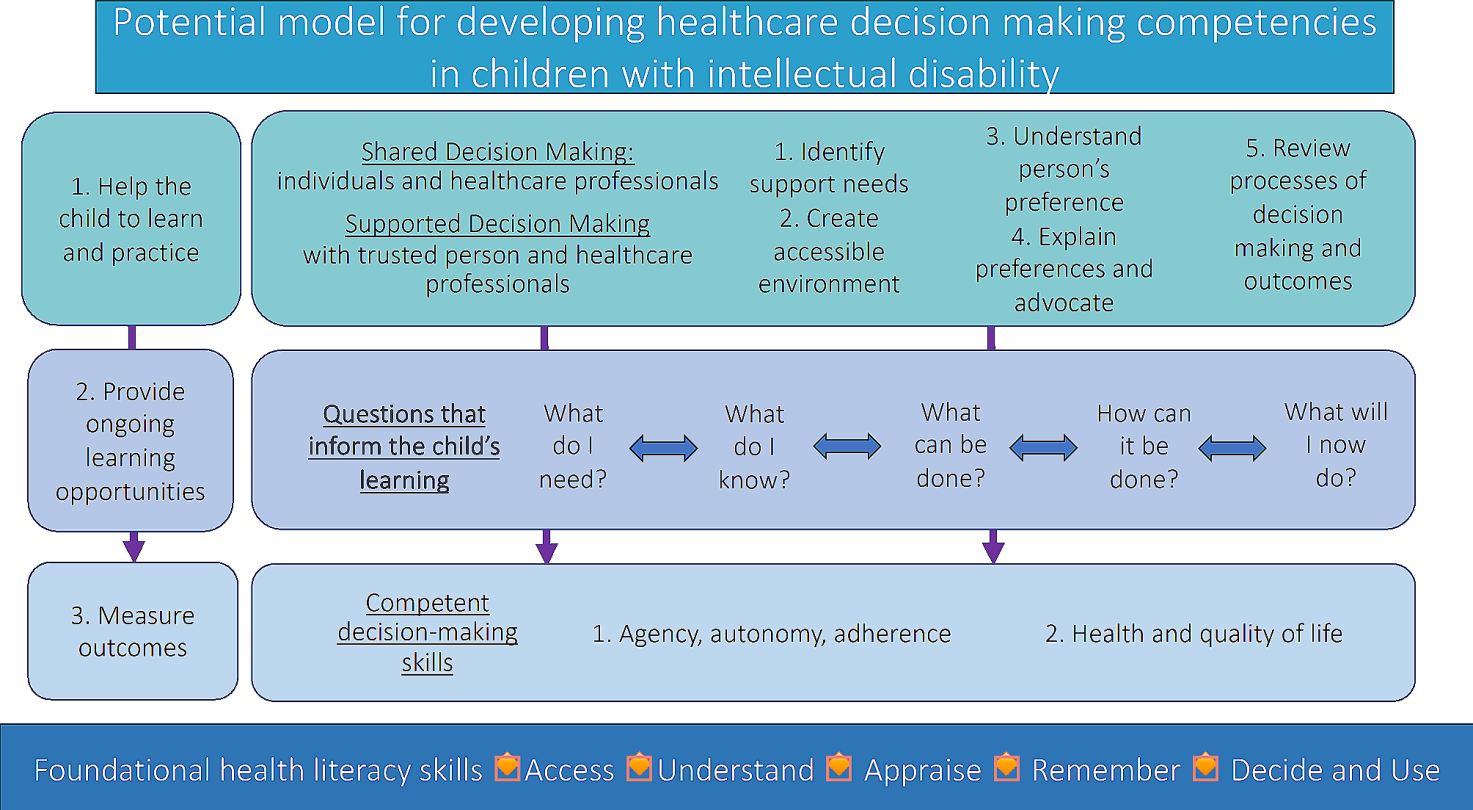
Developing competency in decision making for children and adolescents with intellectual disability. *Footnote:* Children and adolescents with intellectual disability can practice and learn decision-making competencies during shared and/or supported decision-making experiences with potential for improved health outcomes. Frameworks for shared and supported decision making are related and can overlap. The foundational health literacy skills are each intertwined with the skill of decision-making

Whilst most people seek support from healthcare professionals and/trusted individuals for healthcare decision making, children and adults with intellectual disability will need additional supports. Individual capacity to make healthcare decisions will vary, depending on the child’s age and the decision being made, such as its complexity and the risk of the potential outcomes. There are two main approaches to decision making for individuals with intellectual disability: shared decision making and supported decision making [41, 42]. Figure 1 presents how shared and supported decision making may support the child’s learning and practise of skills, with potential to improve health outcomes.

#### Shared decision making

Shared decision making occurs when the individual (i.e., child and/or family) and healthcare professional collaborate throughout the decision-making process to arrive at a plan that aligns with the individual’s values and preferences [32]. Decisions are made with two or more parties, information is shared bi-directionally, and each party is informed and valued equally [32]. Shared decision making is central to Australian healthcare standards [30] including for paediatrics and disability healthcare [32, 35, 43, 44].

For children with chronic illness, shared decision making has been conceptualised as the healthcare professional engaging in *choice talk* (presenting different options, empowering the individual), *option talk* (being aware of recent relevant literature, presenting information in an accessible manner, and avoiding influential language), *decision talk* (discussing individual preferences, comparing short and long-term impacts of the choices, directing individuals to peer-support), and *acceptance of the final decision* made by individual/family [45].

More specific supportive strategies have been described for adolescent healthcare, including *preparation* (adolescent understands their condition, is prepared for the appointment), *communication* (clinician addresses the adolescent directly, engages in one-to-one discussion, encourages the adolescent to lead interactions and share their opinions), and *support* (clinician facilitates opportunities for peer support, builds rapport, demonstrates interest in the adolescent beyond their illness) [46]. While these frameworks suggest a linear process, the reality of shared decision making is that it is iterative with different levels of involvement by the child or adolescent for different components of the healthcare.

A USA study of 2009/10 National Survey of Children with Special Health Care Needs data suggested that shared decision making was more consistently achieved by parents with a child with asthma than those with attention deficit/hyperactivity disorder or autism spectrum disorder [47]. A recent scoping review explored facilitators and barriers to shared decision making with parents for children with complex medical needs, including children with severe neurodevelopmental disability [48]. Commonly reported barriers related to uncertainty about the child’s diagnosis, prognosis or management options, language barriers or poor parent health literacy, power imbalance between clinicians and parents, and lack of continuity in care [48]. In contrast, commonly reported facilitators of shared decision making including valuing the personhood of the child, availability of accessible information, clinician empowerment of parents who then feel comfortable with their decision making, and access to peer support [48]. Literature is lacking on how children with intellectual disability share decision making with their parents or healthcare professionals across the spectrum of intellectual disability.

#### Supported decision making

Supported decision making with people with intellectual disability occurs when the individual works with a trusted person (e.g., family member, friend) to assist them in making their own decisions [33]. Supported decision making enables people with intellectual disability to participate in decisions that affect them, to decide on daily living and participation [33, 49] and healthcare [41, 44, 50] options.

Models describing supported decision making include multiple strategies [44, 49, 50]. As an overview, the support person needs to (1) understand the areas where support is needed (e.g., the level of impairment, type of decision), (2) identify how to support the person with intellectual disability (e.g., having accessible information and effective communication methods), and (3) understand how they will work together to facilitate genuine participation in decisions. Additional strategies include assistance to prepare for appointments, creating an accessible environment at the appointment [50, 51] and the application of a whole-of-organisation culture of engagement with supported decision-making processes [44, 49, 52].

#### Overlap between shared and supported decision making

Shared and supported decision making are not mutually exclusive activities because not all healthcare decisions are made with a healthcare professional present. Further, health literacy skills may promote involvement in both supported and shared decision making. Irrespective of whether shared or supported decision-making is used, guidance for healthcare decision making is needed for a spectrum of healthcare decisions. This might include simple decisions about a blood draw (e.g., when, where, which arm) or deciding whether to undergo a painful and potentially risky procedure, with input titrated to the child’s age and level of intellectual disability.

### Efficacy of shared and supported decision-making strategies – a rapid review

Research exploring the efficacy of shared decision making is primarily situated within the adult medicine and psychiatric literature [53]. However, a scoping review of shared decision making for managing chronic illness in children found seven intervention studies with a control group, including one that used random allocation, and two case series. Participants had neuromuscular scoliosis, allergen immunotherapy, depression, juvenile idiopathic arthritis, obesity, type 1 diabetes, or asthma. Varied outcomes were evaluated in each study providing evidence of improved disease knowledge, reduced decisional conflict and greater satisfaction with health care [43].

**Fig. 2:**
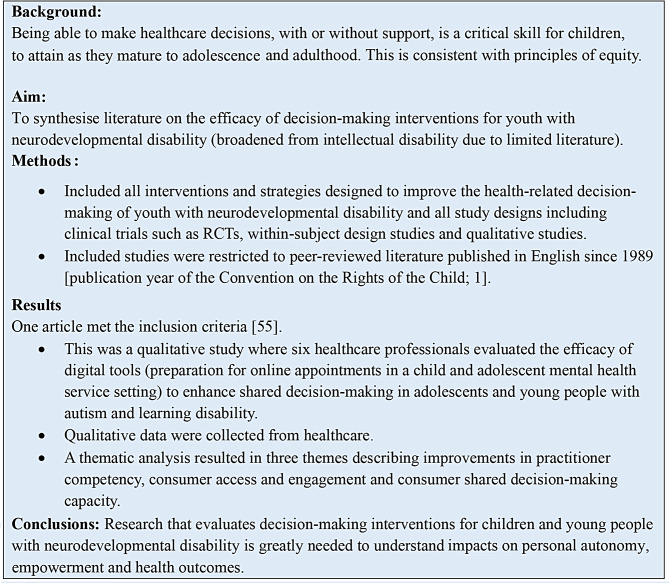
Summary of rapid review (PROSPERO 2023 CRD42023456071)

We undertook a rapid review to synthesise literature on the efficacy of decision-making interventions for children, adolescents and youth with neurodevelopmental disability, searching for intervention studies that used any study design. The initial scoping of the literature suggested limited findings on children with intellectual disability, so we broadened our original scope to include youth with neurodevelopmental disability because there could be important learnings. Figure 2 and **Additional File 1** provide an overview of the rapid review methods which were guided by Cochrane Rapid Review Guidelines [54]. Figure 2 presents a summary of methods and results of the rapid review. **Additional File 1** presents detail of the search strategy, PRISMA flow chart, data extraction and quality assessment.

#### Rapid review result

As presented in Fig. 2 and **Additional File 1**, only one study met the inclusion criteria [55]. This was a qualitative evaluation of an easy read online *shared decision-making tool* used in a child and adolescent mental health service setting for discussing the needs and management of neurodevelopmental assessments for adolescents and young people with autism and learning disability. In this study, the online tool was evaluated from the perspective of the healthcare professionals and not from the perspectives of the adolescents and their families. The healthcare professionals identified the online tool as supporting access to services, encouraging collaborative decision-making, and increasing autonomy of the adolescent patients [55]. The risk of bias (quality) assessment was assessed using the Mixed Methods Appraisal Tool [56]. Although the study had clear research questions and used sufficient data analysis techniques, key methodological weaknesses were identified. Specifically, data were collected from three practitioners participating in a focus group and from another three practitioners who provided written feedback, and the perspectives of the adolescents were not described.

We did not identify any literature that evaluated the effectiveness of *supported* healthcare decision-making interventions for children and young people with a neurodevelopmental disability, including intellectual disability. However, one study that *did not* meet our eligibility criteria because it did not evaluate supported healthcare decision but had relevance to the research question. Eighteen parents of adults with intellectual disability (12 adults with intellectual disability aged younger than 25 years) were trained to support their adult child’s decision making on daily tasks and participation [57]. Qualitative evaluation of parents suggested that they valued the opportunity to reflect and re-evaluate their own perspectives on their adult child’s capacity for decision making, took a more deliberate approach to supporting decision making, and observed their adult child expressing their preferences with greater confidence [57]. This suggests that greater involvement in decision making generally could be associated with benefits to the person with intellectual disability and for their supporter, and informs future approaches and research on supporting healthcare decision making. This is consistent with the findings and recommendations of the recent Australian Disability Royal Commission which will now be discussed.

### The Australian disability royal commission: health literacy and decision-making for children with intellectual disability

Based on literature review and contemporary community expectations, many of the recommendations from the recent Australian Disability Royal Commission were directly relevant to decision making so that individuals with disability are enabled to have greater power in the decisions that affect their life. The recommendations in Volume 6 of the 12-volume final report, titled ‘Enabling autonomy and access’ reflect the concepts of health literacy and healthcare decision making. Table 1 documents the recommendations on components of health literacy including decision making [21]. Interventions and evaluations are needed to ensure their application with children with intellectual disability, their families, clinicians and organisations.

**Table 1:** Selected recommendations from Volume 6 on ‘Enabling autonomy and access’ from the Disability Royal Commission mapped to the World Health Organization’s health literacy actions [21]

Select recommendations	Health Literacy Actions
Recommendation 6.1: A national plan to promote accessible information and communications	Access
Recommendation 6.3: Access to appropriately skilled and qualified interpreters	Access
Recommendation 6.6: Supported decision-making principles	Access, understand, appraise
Recommendation 6.8: Formal supporters	Access, understand, appraise, remember, use
Recommendation 6.10: Decision-making process	Access, understand, appraise, remember, use
Recommendation 6.11: Guidelines on maximising participation	Access, understand, appraise, remember, use
Recommendation 6.13: Information and education on supported decision-making	Training for supporters, clinicians
Recommendation 6.14: Systemic advocacy to promote supported decision-making	Advocacy for supporters, clinicians, organisations, policy makers
Recommendation 6.25: Expand the scope of health workforce capability development to include all forms of cognitive disability at all stages of education and training	Training for supporters, clinicians, organisations, policy makers
Recommendation 6.31: Embed the right to equitable access to health services in key policy instruments	Advocacy targeting organisations, policy makers
Recommendation 6.34: Introduce disability health navigators to support navigation of health care for people with disability	Training targeting supporters, clinicians, organisations, policy makers

Of note, Recommendation 6.6 states that all individuals have an equal right to make decisions, that decision-making capacity is presumed for everyone, and that all people are to be treated with dignity and supported to take risks to live their lives in the way they choose. These are consistent with rights frameworks for children and disability [1, 40]. The critical roles of informal supporters and advocates are recognised and utilised, consistent with contemporary healthcare models for vulnerable individuals [32, 33]. The development and delivery of co-designed policies and practices are consistent with contemporary frameworks for healthcare development [58]. Recommendation 6.10 acknowledges the importance of assisting the person in developing their decision-making abilities, which is critical for all people with disability including children who are developing these skills in preparation for adulthood [38]. Recommendation 6.34 recommends the introduction of disability health navigators as potential enablers of child and family health literacy more broadly, and supporting the skills of accessing, understanding, appraising, and using health information [21, 24].

## Conclusions

Existing rights frameworks and standards indicate the importance of providing all people with the opportunity to learn and practise health literacy skills including healthcare decision making for autonomy. These frameworks were re-iterated in recommendations from the recent Disability Royal Commission in Australia where people with disability want and need greater autonomy. Health literacy is a modifiable determinant of health outcomes and healthcare decision making is inherent in using health literacy skills. As identified in the rapid review, there is very limited literature examining the effectiveness of interventions for healthcare decision making for children and adolescents with intellectual disability and their families, despite this being a critical time for the development of skills and capacity for use during adulthood. The recommendations of the Disability Royal Commission in Australia are contemporary and challenge healthcare professionals to expect disability consumers’ involvement in healthcare decision making and to identify how this can be enabled and evaluate its effectiveness.

As part of multi-level reforms and policy changes that are needed to reduce the social disadvantages experienced by people with intellectual disability [19], evidence for health literacy and decision making interventions for children and adolescents with intellectual disability is also needed to improve the delivery of healthcare and health outcomes. Research is needed to investigate the scope of opportunities suitable for children with different ages and levels of intellectual disability and examine the perspectives and shared roles of children and adolescents with intellectual disability, parents and healthcare professionals. One goal is to develop training protocols for use by families, caregivers, clinicians, educators, and other service providers to teach children with intellectual disability healthcare decision-making skills in preparation for their optimal autonomy in adulthood.

### Electronic supplementary material

Below is the link to the electronic supplementary material.

Supplementary Material 1

## Data Availability

No datasets were generated or analysed during the current study.
