# Supplementary material for: Perspectives on the essential skills of healthcare decision making in children and adolescents with intellectual disability
Source: Int J Equity Health. 2024 Jun 7;23:119. doi: 10.1186/s12939-024-02204-5 (PMC11162048; doi:10.1186/s12939-024-02204-5)
Supplement: Supplementary file 1 — Supplementary Material 1 [file 12939_2024_2204_MOESM1_ESM.docx]

**Summary of Rapid Review Methods and Results**

**1: Research question**

How effective are health related decision-making interventions for children, adolescents and young people (aged 12 to 25 years) with neurodevelopmental disability?

**2: Search Strategy**

**Databases**

CINAHL, Embase, PsycINFO, Cochrane Database Systematic Reviews and ProQuest.

**Search terms**

| Concept 1: Decision making | "decision making" OR decision-making OR "decision support" OR self-management OR "personal management" OR "patient involvement" |
| --- | --- |
| Concept 2: Age | child* OR adolescen* OR "young people" OR youth OR teenage |
| Concept 3: Disability | Disabilit* OR disabled OR "intellectual disabilit*" OR "intellectually disabled" OR "learning disorder*" OR "learning disabilit*" OR "learning dysfunction*" OR "neurodevelopmental disorder*" OR "neurodevelopmental disabilit*" OR "developmental disorder*" OR "developmental disabilit*" OR "developmental delay*" OR retard* OR "mental* handicap*"  OR epilep* OR seizure* OR convulsion* OR Autis* OR ASD OR "child Development Disorder*" OR Asperger* OR ADHD OR ADD OR "attention deficit" OR hyperactive* OR hyperkin* OR "minimal brain dysfunction*" OR "minimal brain damage" OR MBD OR "cerebral palsy" OR "spastic diplegi*" OR "little* disease" OR syndrome* OR "fetal alcohol" OR FASD* OR FAS OR "tic disorder*" OR kernicterus OR "muscular dystroph*" OR deaf* OR blind* OR "hearing loss" OR "hearing impair*" OR "vision disorder*” OR "sensory impair*" OR "vision impair*" OR "low vision" OR "rare disease*" OR "rare diagnos*" OR "rare disabilit*" OR "orphan disease*" OR "orphan disabilit*" OR "orphan diagnos*" OR "motor skills disorder*" OR "developmental coordination disorder*" OR "developmental coordination disorder" OR in-coordination OR dys-coordination OR "minor neurological dysfunction*" OR "motor delay disorder*" OR "perceptual-motor impairment*" OR "motor coordination difficult*" OR "motor learning difficult*" OR "mild motor problem*" OR "motor coordination problem*" OR "sensorimotor difficult*" OR "sensory integrative dysfunction*" OR "psychomotor disorder*" OR "motor control and perception" OR apraxia* OR dyspraxia OR "perceptual motor dysfunction*" OR "minimal cerebral dysfunction" |
| Concept 4: Health | health* OR "mental health" OR depressi* OR anxi* |
| Concept 5: Study design | RCT OR review OR "before and after" OR before-after OR "single subject" OR single-subject OR "within subject" OR intervention* OR trial* |

**3: Inclusion/Exclusion Criteria**

| **Information** | **Include** | **Exclude** |
| --- | --- | --- |
| Topic | Evaluation of the efficacy of decision-making interventions for children and adolescents and youth with a neurodevelopmental disability. | Studies that do not include all of these factors. |
| Sample | Young people aged between 12 to 25 years with a neurodevelopmental disability. Do not exclude if the study sample does not include this entire range.  Do not exclude if the study's sample is beyond the age range but data related to the age range is extractable.  The mean and standard deviation values need to be within the age range. | Sample is younger than 12 and/or older than 25 years.    The sample includes participants who are both within and significantly beyond the age range (mean value outside 12 to 25 years of the mean value is within 12 to 25 years but one SD value is outside the 12-to-25-year range).  Specific data relating to the included age range cannot be identified. |
| Publication date | Literature published from  1989, when the  Convention on the Rights of the Child was ratified. | Literature published before 1989. |
| Data type | Any quantitative, qualitative and mixed methods peer-reviewed literature that evaluates the efficacy of decision-making interventions for adolescents with neurodevelopmental disability. | Grey literature: Books, dissertations, research and government reports, conference papers, ongoing research, media content (e.g., newspaper, magazines), letters to editors, editorials, blogs, podcasts, newsletters, wire feeds |
| Language | Published in English | Not published in English |
| Study design | All quantitative, mixed methods and qualitative evaluation studies that assess the outcome of decision-making interventions for children, adolescents and young people with neurodevelopmental disability. | NA |
| Intervention | All intervention types included. | NA |
| Outcome | All outcomes of evaluations are included even if efficacy was shown to be poor. | NA |
| Quality | Peer-reviewed quantitative data will not be excluded due to poor quality. | NA |
| Availability | Included if the full text is available through the institution. | Full text is not available through the institution. |

**4: PRISMA Flowchart**

Identification of studies via database searching

Identification

Duplicate records removed before screening

(*n* = 900)

Records identified from databases

(*n* = 1903)

Records excluded

(manually)

(*n* = 996)

Screening

Records screened

(title and abstract review)

(*n* = 1003)

Studies excluded

(e.g., intervention not evaluated, intervention did not targeted health literacy skills or youth with neurodevelopmental disability)

(*n* = 6)

Studies assessed for eligibility

(full-text review)

(*n* = 7)

Included

Articles included in the final review

(*n* = 1)

Note. All studies identified for full text review were retrieved.

| Study, Country | Research design | Participant characteristics | Medium of intervention | Intervention | Comparison | Outcomes | Measures | Timing of assessment(s) | Results |
| --- | --- | --- | --- | --- | --- | --- | --- | --- | --- |
| Bates et al 2021  UK | Qualitative quality improvement project involving online focus groups and written feedback. | 6 practitioners e.g., clinical, psychologist, psychological therapist.  An unspecified number of young people with autism/learning disability and their parents provided initial feedback on decision making aids. However, these data are not reported. | Decision aid booklets. | Two booklets were created to assist young people with autism/learning disability to make health-care decisions. One booklet aimed to aid in decision making around online psychological appointments. The other booklet aimed to aid in decision making around completing an autism assessment. | No comparison. | Practitioner experience using decision-making aid, including feedback on youth's perspectives and areas for improvement in using aid. | No outcome measures.  Responses to focus groups were coded thematically. | Not specified. | Three findings emerged from the thematic analysis:  1. The guides facilitated greater engagement with and promotion of young people’s independence in collaborative decision-making  and providing informed consent.  2. Parents/carers of adolescents and young adults with Autism were considered to have benefited from having the space to consider their child’s right to privacy and their progress towards more independent decision-making  3. The guides helped the practitioners build their competence in navigating and explaining online appointments. |

**5: Data Extraction Table**

**6: Quality Assessment using the Mixed Methods Appraisal Tool (MMAT)[1]***

| S1. Are there clear research questions? | S2. Do the collected data allow to address the research questions? | 1.1. Is the qualitative approach appropriate to answer the research question? | 1.2. Are the qualitative data collection methods adequate to address the research question? | 1.3. Are the findings adequately derived from the data? | 1.4. Is the interpretation of results sufficiently substantiated by data? | 1.5. Is there coherence between qualitative data sources, collection, analysis and interpretation? |
| --- | --- | --- | --- | --- | --- | --- |
| **Y** | **Can’t tell** | **Can’t Tell** | **N** | **Y** | **Y** | **Can’t tell** |

* We used the Mixed Methods Appraisal Tool (MMAT) scale for evaluating the quality of selected references in this study because the inclusion criteria included any study design. Each scale in the MMAT has two general screening questions (S1 and S2) and five unique quality assessment criteria, which are assessed using three response options (yes, no, cannot tell). The MMAT subscale for qualitative studies was used to assess study risk of bias (quality) of the sole paper identified in the rapid review.

1. Hong QN, Pluye P, Fàbregues S, Bartlett G, Boardman F, Cargo M, Dagenais P, Gagnon M-P, Griffiths F, Nicolau B, et al: **Mixed Methods Appraisal Tool (MMAT) Version 2018: User guide.** pp. 1356-1294. Montreal, Canada: McGill; 2018:1356-1294.
